# The Shelterin TIN2 Subunit Mediates Recruitment of Telomerase to Telomeres

**DOI:** 10.1371/journal.pgen.1005410

**Published:** 2015-07-31

**Authors:** Amanda K. Frank, Duy C. Tran, Roy W. Qu, Bradley A. Stohr, David J. Segal, Lifeng Xu

**Affiliations:** 1 Department of Microbiology and Molecular Genetics, University of California, Davis, Davis, California, United States of America; 2 Department of Pathology, University of California, San Francisco, San Francisco, California, United States of America; 3 Genome Center and Department of Biochemistry and Molecular Medicine, University of California, Davis, Davis, California, United States of America; Chinese Academy of Sciences, CHINA

## Abstract

Dyskeratosis Congenita (DC) is a heritable multi-system disorder caused by abnormally short telomeres. Clinically diagnosed by the mucocutaneous symptoms, DC patients are at high risk for bone marrow failure, pulmonary fibrosis, and multiple types of cancers. We have recapitulated the most common DC-causing mutation in the shelterin component TIN2 by introducing a TIN2-R282H mutation into cultured telomerase-positive human cells via a knock-in approach. The resulting heterozygous TIN2-R282H mutation does not perturb occupancy of other shelterin components on telomeres, result in activation of telomeric DNA damage signaling or exhibit other characteristics indicative of a telomere deprotection defect. Using a novel assay that monitors the frequency and extension rate of telomerase activity at individual telomeres, we show instead that telomerase elongates telomeres at a reduced frequency in TIN2-R282H heterozygous cells; this recruitment defect is further corroborated by examining the effect of this mutation on telomerase-telomere co-localization. These observations suggest a direct role for TIN2 in mediating telomere length through telomerase, separable from its role in telomere protection.

## Author Summary

The shelterin complex protects telomeres from being processed by the DNA damage repair machinery, and also regulates telomerase access and activity at telomeres. The only shelterin subunit known to promote telomerase function is TPP1, which mediates telomerase recruitment to telomeres and stimulates telomerase processivity. Mutations in shelterin components cause Dyskeratosis Congenita (DC) and related disease syndromes due to the inability to maintain telomere homeostasis. In this study, we have identified TIN2-R282H, the most common DC-causing mutation in shelterin subunit TIN2, as a separation-of-function mutant which impairs telomerase recruitment to telomeres, but not chromosome end protection. The telomerase recruitment defect conferred by TIN2-R282H is likely through a mechanism independent of TIN2’s role in anchoring TPP1 at telomeres, since TPP1 localization to telomeres is unaffected by the mutation.

## Introduction

The multi-subunit shelterin complexes bind along mammalian telomeres, shielding the natural chromosome ends from engaging the DNA damage signaling and repair machinery [1]. Among the shelterin components, TRF1 and TRF2 bind directly to duplex telomeric repeats [2], while POT1 binds to the single-stranded regions of telomeres [3]. TPP1 forms a heterodimer with POT1 and enhances the affinity of POT1 for telomeric ssDNA [4]. Depletion of TPP1 or POT1 results in the deregulation of the single-stranded telomeric terminal overhang and the induction of a DNA damage response at telomeres [5–7]. TIN2 directly interacts with TRF1, TRF2 and TPP1, assuring structural integrity of the complex [8–10]. Depletion of TIN2 causes profound telomere deprotection phenotypes including destabilization of the shelterin complex, activation of telomeric DNA damage signaling, and increased apoptosis [9,11–14].

Increasing evidence suggests that the shelterin complex also regulates access of telomerase to telomeres and hence telomerase action on them. The best evidence for a shelterin-specific role in telomerase regulation comes from analysis of TPP1, which interacts with the telomerase catalytic subunit through the N-terminal OB-fold domain of TPP1 [15–18]. This interaction is crucial for recruiting telomerase to telomeres, as assessed by co-localization of telomerase RNA to telomeres through *in situ* hybridization analysis [19]. The TPP1/POT1 heterodimer also promotes telomerase processivity, as demonstrated by an *in vitro* direct telomerase activity assay [4,20]. Notably, mutations in the TPP1 OB-fold domain compromise telomerase-dependent telomere extension but not telomere end protection [18,21], indicating that TPP1 performs a role in telomerase regulation which is distinct from its contribution to chromosome end protection.

Whether other shelterin components also directly contribute to telomerase regulation has been less well characterized. Depletion of TIN2, which associates with TPP1, leads to reduced levels of TPP1-mediated telomerase association to telomeres [19], although this result might simply reflect an indirect function for TIN2 as a regulator of telomerase recruitment through anchoring TPP1 at telomeres. Intriguingly, an N-terminally truncated form of TIN2 lacking the TPP1 interaction domain can still induce significant telomerase-dependent telomere extension [8,22], suggestive of a TPP1-independent role for TIN2 in telomerase regulation.

An important resource for genetic defects in both telomerase and shelterin has come from Dyskeratosis Congenita (DC) patients. DC is an inherited disorder caused by abnormally short telomeres [23]. Clinically diagnosed by the mucocutaneous abnormalities, DC patients are prone to developing bone marrow failure, multiple types of cancers and a spectrum of diseases collectively characterized as “telomere syndromes” [24]. DC-causative mutations have been found in various telomerase ribonucleoprotein components affecting enzymatic activity (*i*.*e*. the telomerase catalytic subunit TERT and the RNA subunit TER) as well as telomerase biogenesis and trafficking (*i*.*e*. Dyskerin, NHP2, NOP10 and TCAB1) [25–30]. Recently, additional DC-causative mutations have been identified in shelterin components (*i*.*e*. TIN2 and TPP1) and other proteins involved in telomere replication (*i*.*e*. RTEL1 and CTC1) [31–38]. TIN2 mutations in DC patients correlate with aberrantly shortened telomeres and early onset of DC. Almost all patients reported thus far are heterozygotes, harboring only one mutated allele of TIN2. The vast majority of the disease-related TIN2 mutations are missense point mutations that cluster in a highly conserved yet uncharacterized region in the TIN2 C-terminus (a.a. 280–291), outside of the known TRF1, TRF2 or TPP1 interaction regions [10,39], with R282H being the most frequently observed mutation.

Using an ectopic expression system to examine the consequences of TIN2 DC mutations on telomere length in human cells, one recent study reported that overexpression of TIN2 DC mutants caused accelerated telomere shortening in HT1080-derived HTC75 cells, through a telomerase-dependent pathway [40]. However, another study challenged these conclusions and reported that overexpression of wild-type TIN2 and TIN2 DC mutants produced indistinguishable telomere length changes in HT1080 cells [41]. We note that overexpression studies have serious limitations as models to characterize the mechanistic basis for TIN2 dysfunction in DC patients: First, as observed by one of the above studies [40], ectopic expression of TIN2 increased endogenous TRF1 and TPP1 levels (both of which have roles in telomere length regulation). In contrast, neither TPP1 nor TRF1 accumulates to higher levels in TIN2 DC cells (under conditions where the mutant TIN2 protein is expressed at endogenous levels) than in wild-type cells. Second, since TIN2 binds to multiple shelterin proteins but not directly to telomeric DNA, overexpression of TIN2 can potentially sequester other shelterin proteins from telomeres. These side effects complicate the interpretation of TIN2 overexpression studies and may have caused the discrepancy between the overexpression studies. Collectively, these results have left unaddressed whether TIN2, like TPP1, has a direct role in telomerase regulation that can be distinguished from its telomere end protection activity.

Here we recapitulate TIN2-R282H heterozygosity in cultured telomerase-positive human cells using a zinc finger nuclease mediated knock-in approach to generate a TIN2 DC allele expressed at its normal level from its endogenous locus. Unlike the DC patient-derived cells which have been carrying the TIN2 mutations for years and may have acquired secondary compensatory mutations or epigenetic changes that potentially complicate the interpretation of phenotypes, our approach allows analysis of the immediate telomere phenotype in isogenic human cell clones that differ only in their TIN2 status. Analyses of these cells demonstrate that the TIN2-R282H heterozygosity has no impact on the telomere protection function of TIN2. Instead, we show that this separation-of-function defect in TIN2 leads to impaired telomerase recruitment, resulting in a reduced frequency of telomerase-mediated telomere extension events. These observations identify a second subunit of shelterin that mediates telomerase function, thereby further extending the premise that shelterin performs a dual role at telomeres.

## Results

### A common DC-causative mutation in TIN2 results in progressive telomere shortening

Zinc finger nuclease (ZFN) mediated gene targeting was used to knock a TIN2 DC mutation into the human colon carcinoma cell line HCT116. HCT116 cells were chosen because they have active telomerase and wild-type shelterin components, they maintain a stable diploid karyotype suitable for gene targeting, and they are intact for most DNA damage-dependent checkpoints [42]. To knock TIN2 DC mutations into HCT116 cells, we designed a pair of ZFNs that specifically recognize unique sequences within TIN2 exon 6 [43] (S1A and S1B Fig). Two donor template constructs were used in the knock-in study: one carries a G to A mutation within exon 6 to introduce a single amino acid change from Arg to His at position 282 (R282H, one of the most frequently observed TIN2 mutations in DC patients); the other carries the wild-type sequence (WT) to generate the isogenic wild-type control cells. Translational silent mutations were introduced into the donor template at the ZFN recognition sites to prevent binding and cleavage of the construct by the ZFN pair.

Two knock-in clones heterozygous for the TIN2-R282H mutation (clone R282H.1 and R282H.2) and two knock-in clones wild-type for the TIN2 gene (clone WT.1 and WT.2) were established (S1C, S1D, and S1E Fig; see also Materials and Methods for details). Two TIN2 splice variants had been previously identified in human cells. The R282H mutation lies in the middle of the sixth exon shared between the splice variants (S1B and S2A Figs). As shown in S2B Fig, the expected TIN2 splice variants were produced in both the HCT116 parental cells and the knock-in clones, and sequencing of the reverse transcription products confirmed that both the mutated allele and the wild-type allele were transcribed into mRNA in the TIN2-R282H heterozygotes (S2C Fig). The functional difference between the two TIN2 splice variants is not yet characterized. In multiple human cell lines, only TIN2S could be detected [14,44,45], possibly due to the low abundance of the TIN2L protein. Immunoblotting analysis with an anti-TIN2 antibody raised against an N-terminal epitope of TIN2 (a.a. 44–58) detected one TIN2 protein band (TIN2S) of ~42KD in all clones (S2D Fig), consistent with the other reports. TIN2 protein levels in the R282H heterozygous clones were indistinguishable from those in the WT clones and parental HCT116 cells (S2D Fig), demonstrating that the R282H mutation did not significantly change TIN2 protein stability. Levels of the other five shelterin proteins were indistinguishable between TIN2-WT and TIN2-R282H clones as well (S2E Fig). Furthermore, immunoprecipitation analysis showed that there was no change in the interaction between TIN2 and its shelterin binding partner TRF1, TRF2 or TPP1 in the TIN2-R282H heterozygotes (S2F Fig).

**Fig 1:**
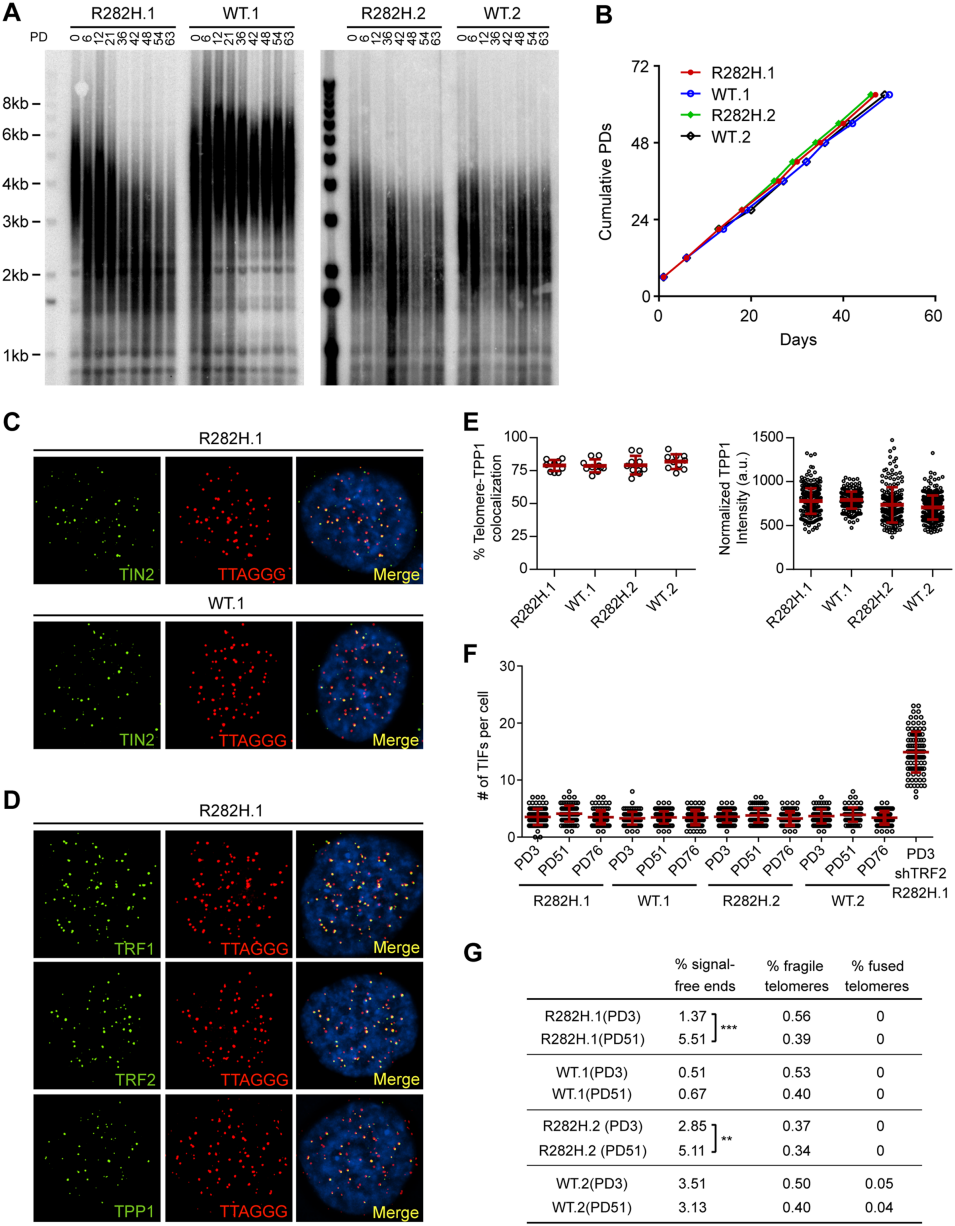
Heterozygous TIN2-R282H mutation induces progressive telomere shortening, but not gross telomere deprotection, in HCT116 cells. (**A**) Telomeres were maintained in cells with wild-type TIN2, but progressively shortened in cells with heterozygous TIN2-R282H mutation. Cells were continuously passaged and collected at the indicated population doublings (PD). Genomic DNAs were extracted and bulk telomere lengths were examined by Telomere Restriction Fragment analysis using a telomeric repeat probe. (**B**) Growth curve of HCT116 knock-in cells. (**C**) Telomeric localization of TIN2 examined by immunofluorescence-FISH analysis in cells carrying heterozygous TIN2 mutation (clone R282H.1, PD8 cells) and wild-type TIN2 (clone WT.1, PD8 cells). For immunofluorescence staining, we used an anti-TIN2 antibody (Imgenex) (produced against an N-terminal epitope of TIN2 (a.a. 44–58)) which recognizes both the wild-type and mutant TIN2. Telomeres were detected by FISH using a PNA telomeric probe. (**D**) Telomeric localization of TRF1, TRF2 and TPP1 examined by immunofluorescence-FISH analysis in cells carrying heterozygous TIN2 mutation (clone R282H.1, PD8 cells). (**E**) Left panel: quantification of fraction of telomeres co-localizing with TPP1. Each circle on the graph represents a single nucleus. Telomeres in 10 nuclei were examined for each knock-in clone. Mean ± SD indicated by red lines. Right panel: TPP1 fluorescence intensity, normalized against telomere length (based on telomere FISH signal intensity). Each circle on the graph represents a single telomere. Mean ± SD indicated by red lines. Telomeres in 10 nuclei were analyzed for each clone. (**F**) Quantification of telomere dysfunction-induced DNA damage foci (TIFs) in early (PD3) and late PD (PD51 and PD76) HCT116 knock-in clones. For positive control, R282H.1 cells were treated with shRNA against TRF2 and TIFs quantified. DNA damage was assessed by immunostaining with an antibody against 53BP1 and telomeres were detected by PNA FISH using a telomeric probe. All quantifications were carried out blindly. Each circle on the graph represents a single cell. Mean ± SD indicated by red lines. TIFs in 100 cells were analyzed for each sample. (**G**) Quantification of telomeric abnormalities from metaphase spreads of HCT116 knock-in clones. Cells were collected at indicated PDs for metaphase spreading followed by FISH analysis using a PNA telomeric probe and a centromeric probe. ~4000 telomeres were analyzed for each sample. All quantifications were carried out blindly.

To examine the effect of the TIN2-R282H mutation on telomeres in cells with active telomerase, we monitored telomere length of the HCT116 knock-in cells over successive cell divisions. Because telomere length is a heterogeneous trait, sub-clones of human cell lines with the same genotype can display variations in mean telomere length, as shown in Fig 1A and previously observed [46]. Despite differences in initial telomere length, telomeres in both TIN2-R282H heterozygote clones shortened progressively until they reached a mean telomere length slightly above 2kb; as expected, telomeres in TIN2-WT cells maintained stable lengths (Fig 1A). This establishes that a primary consequence of the TIN2-R282H heterozygous mutation is a progressive reduction in telomere length that occurs even in the presence of telomerase.

### TIN2-R282H heterozygotes do not exhibit a telomere deprotection defect

Notably, TIN2-R282H heterozygotes had a similar proliferation rate as TIN2 wild-type cells, even at late PDs (Fig 1B). Furthermore, immunostaining analysis showed that there were no detectable changes of telomeric localization for either TIN2 or other shelterin proteins in TIN2-R282H heterozygotes (Fig 1C and 1D). In particular, we found no evidence for TPP1 delocalization in response to the TIN2-R282H heterozygous mutation (Fig 1E), arguing that the telomere length phenotype conferred by the TIN2-R282H defect was not simply due to an indirect effect on TPP1 delocalization.

These initial observations also suggested that telomere protection was not impaired by the TIN2-R282H defect. To assess this more rigorously, we evaluated telomere dysfunction-induced foci (TIF) formation in early and late PD knock-in cells by performing immunostaining with an antibody against the DNA damage marker 53BP1 and telomeric fluorescent *in situ* hybridization (FISH) with a telomeric peptide nucleic acid (PNA) probe. Although HCT116 cells were fully functional for telomere dysfunction-induced DNA damage signaling (as indicated by the localization of 53BP1 to telomeres in HCT116 depleted of TRF2), there was no significant increase of TIFs, even in late PD TIN2-R282H heterozygote cells (PD51 and PD76) (Fig 1F). Together these results show that the heterozygous TIN2-R282H mutation does not cause shelterin redistributions or gross deprotection of telomeres.

TIN2 knock-in cells were also collected at early and late PDs for FISH analysis of metaphase spreads and examined for telomere abnormalities. No significant differences were found between TIN2-WT and TIN2-R282H cells at early population doublings. By PD51, the TIN2-R282H heterozygotes had a statistically significant increase in chromosome ends lacking detectable telomeric signals, which presumably reflects the very short telomeres in these cells. Notably, however, we did not observe an increase in fragile telomeres or chromosome end-to-end fusions (Fig 1G), further supporting the conclusion that TIN2-R282H heterozygosity did not lead to chromosome end deprotection.

### Telomere shortening in TIN2-R282H heterozygotes does not occur via a telomerase-independent pathway

**Fig 2:**
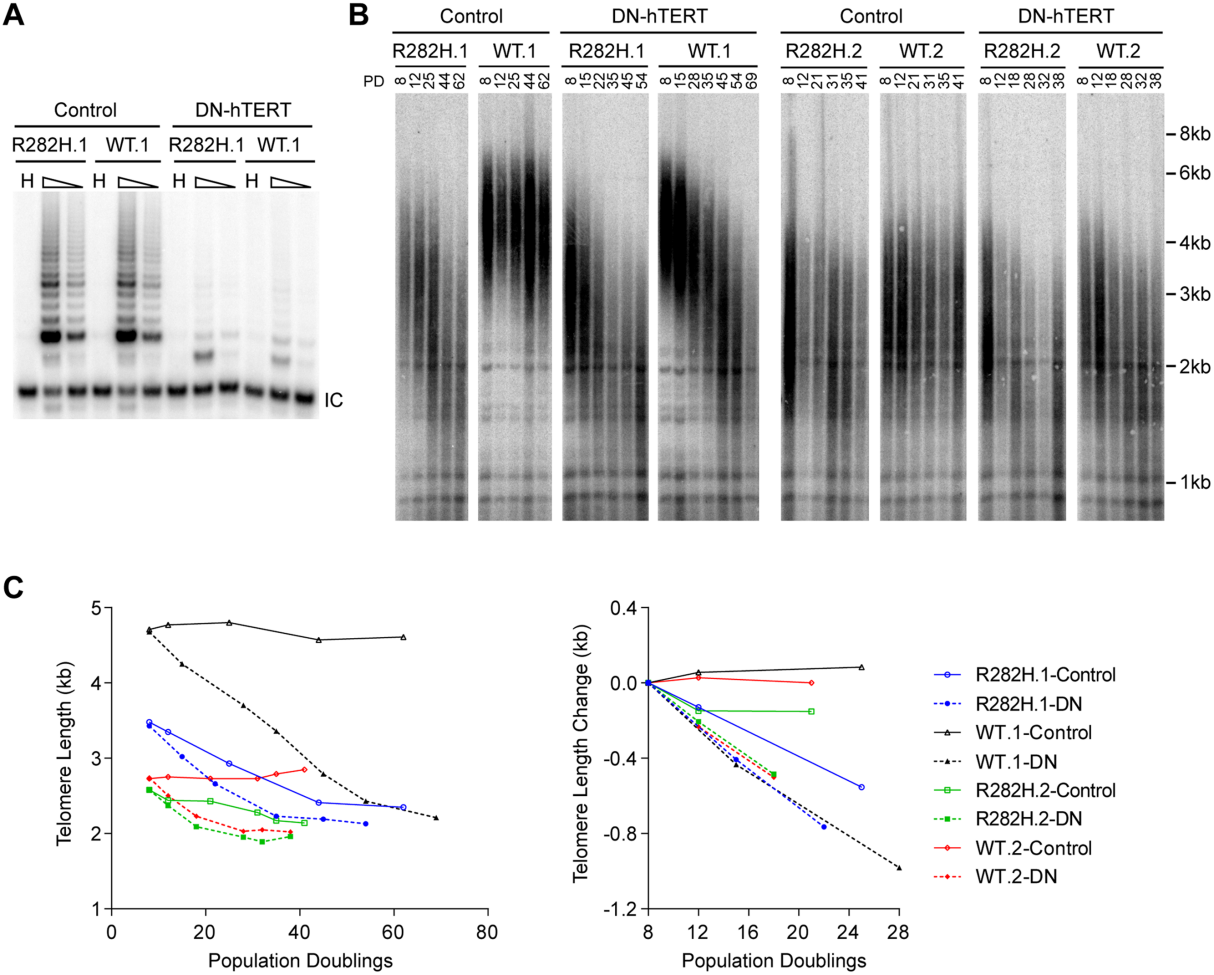
Telomerase inhibition in TIN2-R282H heterozygotes and TIN2-WT cells leads to similar rates of telomere erosion. (**A**) Representative TRAP assay results from HCT116 knock-in clones infected with lentivirus expressing dominant-negative telomerase catalytic subunit (DN-hTERT) or luciferase control. Whole cell extracts from 500 and 100 cells at PD21 were analyzed for each line. (**B**) Telomere Restriction Fragment analysis of HCT116 knock-in clones. Cells from each infection were pooled, continuously passaged and collected at indicated population doublings (PD). (**C**) Left panel: Mean telomere lengths in (**B**) were determined by the ImageQuant software and plotted against PDs. Right panel: Quantification of changes in mean telomere length between PD8–28.

The progressive telomere shortening in TIN2-R282H heterozygotes can be caused by either a defect in the telomerase pathway or by a telomerase-independent process such as increased telomere degradation. To distinguish between these two possibilities, we asked whether combining the TIN2-R282H mutation with a telomerase defect would confer an additive effect on telomere shortening, which would argue that TIN2-R282H mediated its effect on telomere length through a telomerase-independent mechanism. We ectopically overexpressed a dominant-negative form of telomerase catalytic subunit (DN-hTERT) [47] in parallel in TIN2-R282H heterozygotes and TIN2-WT cells (at PD8). DN-hTERT overexpression suppressed telomerase activity to undetectable levels as expected (Fig 2A), and caused progressive telomere shortening in all HCT116 knock-in clones (Fig 2B and 2C). Measurement of telomere length changes between PD8-PD28 cells showed that the expression of DN-hTERT led to similar rates of telomere shortening in TIN2-WT and TIN2-R282H cells (Fig 2C).

### An *in vivo* telomerase activity assay demonstrates that telomerase extends telomeres at a reduced frequency in TIN2-R282H heterozygotes

The above observations led us to consider that the mechanism underlying the progressive telomere shortening in TIN2 heterozygote cells was due to a defect in the telomerase pathway. To address this, we first monitored activity levels of the telomerase enzyme. Quantitative PCR analysis of endogenous telomerase RNA in HCT116 knock-in clones showed that the TIN2-R282H mutation did not cause a significant change in telomerase RNA levels (S3A Fig). Telomerase TRAP analysis also showed that telomerase enzymatic activity in TIN2-R282H heterozygotes was indistinguishable from that in TIN2-WT cells (S3B Fig), indicating that the progressive telomere shortening in TIN2-R282H heterozygotes was not caused by a reduction of the core telomerase enzymatic activity.

**Fig 3:**
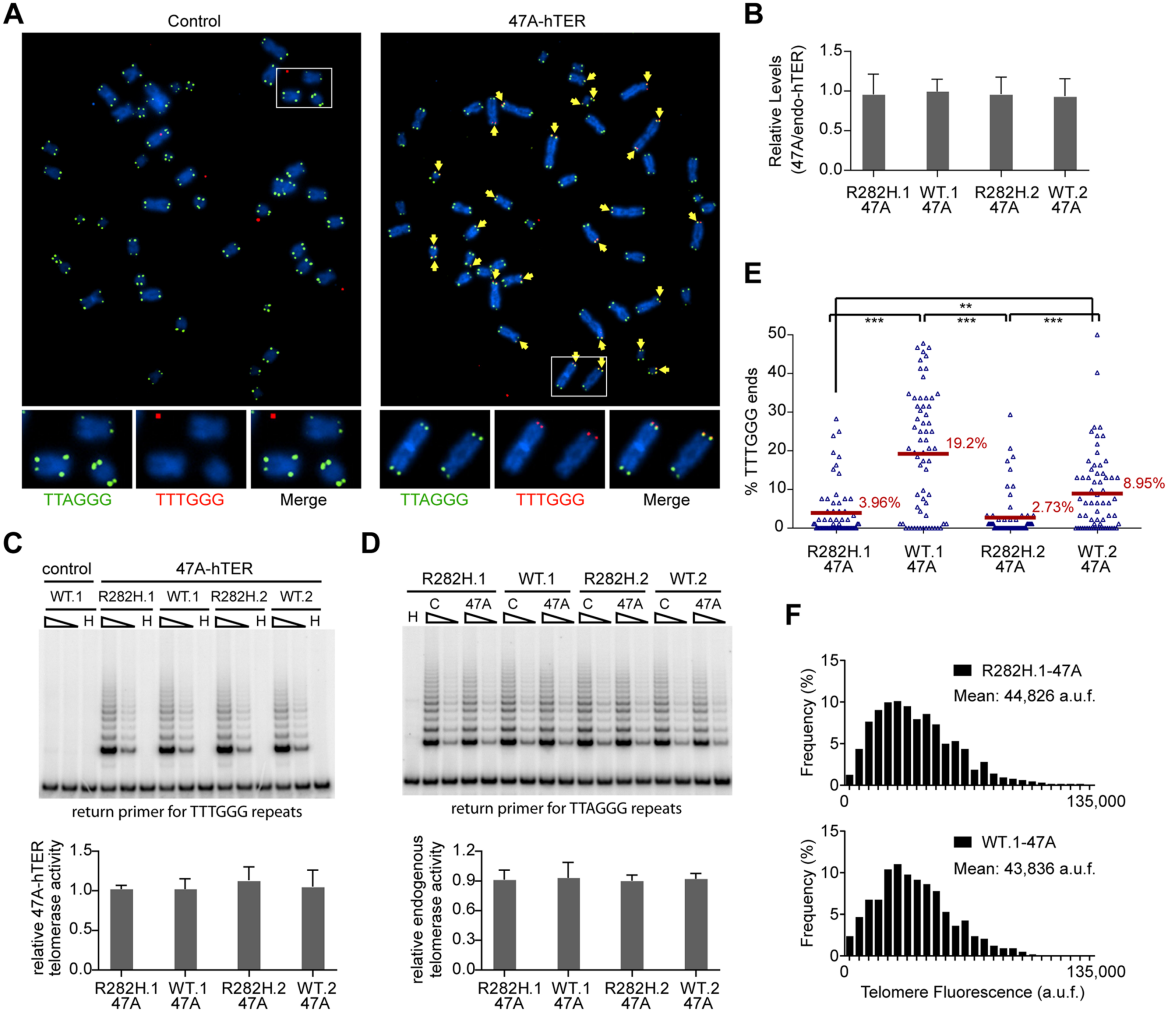
Heterozygous TIN2-R282H mutation decreases the frequency of telomere extension by telomerase. HCT116 knock-in clones were infected with lentivirus expressing 47A-hTER to achieve a 1:1 steady state expression level of 47A-hTER: endogenous-hTER in each clone. 8 days after infection, parallel cultures of cells were collected for metaphase spreads followed by telomeric FISH, for quantitative PCR, for TRAP assay, and for counting of cell numbers. (**A**) Representative telomeric FISH images showing 47A-hTER-directed incorporation of TTTGGG variant repeats at telomeres in WT.1 cells. Cells were infected with an empty lentiviral vector control or lentivirus expressing 47A-hTER. Telomeric FISH was carried out using PNA probes for the canonical TTAGGG repeats (green) and the variant TTTGGG repeats (red). Telomeres incorporating TTTGGG repeats were marked with yellow arrows. Regions encircled in white boxes are enlarged at the bottom of the corresponding image for better visualization. (**B**) Relative steady state expression levels of 47A-hTER determined using QPCR. Expression levels were normalized to GAPDH and relative to respective endogenous telomerase RNA levels in each clone. Bars represent mean values of three experiments and SDs. (**C**) 47A-hTER assembles into equivalent levels of active telomerase in all knock-in clones. Top panel: whole cell extracts were examined for 47A-hTER-containing telomerase activity by 47A-hTER-specific TRAP assay using return primer 5’-GCGCGGTACCCATACCCATACCCAAACCCA-3’. Extracts from 400 and 100 cells were analyzed for each sample. WT.1 cells infected with the empty lentiviral vector was used as control to show the specificity of the TRAP assay conditions. Bottom panel: Quantification of relative TRAP activity of 47A-hTER-containing telomerase in each indicated clone, relative to that in R282H.1–47A cells. Bars represent mean values of three experiments and SDs. (**D**) Endogenous wild-type telomerase activity in knock-in clones infected with an empty lentiviral vector or lentivirus expressing 47A-hTER. Top panel: the same whole cell extracts as described in (C) were examined for endogenous wild-type telomerase activity using return primer 5’- GCGCGGTACCCTTACCCTTACCCTAACCCT-3’. Extracts from 100 and 25 cells were analyzed for each sample. Bottom panel: Quantification of relative endogenous wild-type telomerase activity in each indicated clone, relative to respective clones infected with a lentiviral vector control. Bars represent mean values of three experiments and SDs. (**E**) Quantification of fraction of telomeres incorporating TTTGGG repeats in knock-in clones expressing 47A-hTER. Data were obtained analyzing > 2500 chromosomes in ~60 metaphase spreads from each clone. All quantifications were carried out blindly. Each point on the graph represents a single metaphase spread. Mean values are indicated in red. *** (p≤0.001) ** (p≤0.01) calculated by two-tailed Student’s t-tests. (**F**) Quantification of telomeric TTTGGG fluorescence intensity. The distributions of fluorescence intensities, in arbitrary fluorescence unit, of more than 600 telomeric TTTGGG spots from metaphase spreads of each indicated clone are displayed. All quantifications were carried out blindly.

Since telomerase levels did not appear to be altered, we employed two assays to monitor the extent of telomerase activity (this sub-section) or telomerase recruitment (the next sub-section) at individual telomeres. To directly measure telomerase activity *in vivo*, we designed a novel assay to measure telomerase extension events at individual telomeres, by adapting a FISH-based assay which utilizes a telomerase enzyme that adds variant telomeric repeats to telomeres [48]. When the mutant template telomerase RNA, 47A-hTER, is expressed in telomerase-positive cell lines, it assembles with endogenous telomerase catalytic subunit hTERT into active telomerase to direct the incorporation of TTTGGG variant repeats at telomeres [49–51]. FISH with a (CCCAAA)_3_ PNA probe specifically detects the TTTGGG variant repeats, whereas the (CCCTAA)_3_ PNA probe specifically detects the canonical TTAGGG telomeric repeats (Fig 3A **)**. In prior experiments with the mutant 47A-hTER telomerase RNA, it was detrimental to cell growth, causing chromosome end-to-end fusions [48,50]. However, in these previous experiments, the steady state expression level of 47A-hTER was at a ≥10:1 ratio relative to the endogenous hTER. In contrast, when the 47A-hTER was expressed at low levels (at ~1:1 ratio relative to the endogenous hTER; Fig 3B) for a limited number of cell divisions in HCT116 cells, we did not observe either chromosome fusion or growth inhibition effects (Fig 3A and S4A and S4B Fig), even though mutant TTTGGG repeats were added by telomerase to chromosome termini, as assessed by FISH (Fig 3A **)**. The presence of very low levels of a variant telomerase enzyme allows us to quantify two aspects of telomerase activity at individual telomeres: (i) the relative frequency of telomere extension events, as determined by counting the fraction of telomeres that had incorporated TTTGGG repeats; and (ii) the relative length of extension at individual telomeres, as determined by measuring the fluorescence intensity of TTTGGG repeats at these newly extended telomeres. This provides a powerful assay to directly monitor the activity of telomerase at individual telomeres, at a resolution that has not been previously attainable by other approaches.

Expression of 47A-hTER at a 1:1 ratio relative to the endogenous hTER in each of the knock-in clones (Fig 3B) led to the assembly of enzymatically active 47A-hTER-containing telomerase at comparable levels in the knock-in clones, as shown by the 47A-hTER-specific TRAP analysis in Fig 3C (see Materials and Methods and S5 Fig for TRAP assay conditions that distinguish between 47A-hTER telomerase and wild-type telomerase). TRAP analysis also revealed that the expression of 47A-hTER caused only ~10% decrease of wild-type telomerase activity in each of the knock-in clones (Fig 3D), suggesting that 47A-hTER titrated away ~10% of hTERT from the endogenous telomerase complex. The reconstitution of only a small proportion of endogenous telomerase into 47A-hTER-containing telomerase in this time frame (8 days) likely reflects the extreme stability and very long half-life reported for telomerase RNA in telomerase-positive human cancer cells [52].

Due to the very low levels of the reconstituted 47A-hTER telomerase, only a small subset of telomeres in the knock-in clones had incorporated TTTGGG variant repeats (Fig 3E). Using this assay, we observed that the fraction of chromosome ends incorporating the variant repeats per metaphase in TIN2-R282H heterozygote cells was significantly less than that in TIN2-WT cells (Fig 3E), indicating that the R282H mutation caused a reduction in the frequency of telomere extension events. Strikingly, although the number of chromosome ends elongated by telomerase (based on the incorporation of variant TTTGGG repeats) was reduced in TIN2-R282H heterozygous cells, the amount of TTTGGG repeats added by the 47A-hTER telomerase at individual telomeres was not affected. This was revealed by measuring the fluorescence intensity of the TTTGGG variant repeat tracts at individual termini. As shown in Fig 3F, the distribution of the TTTGGG signal intensity in TIN2-R282H heterozygote cells was indistinguishable from that in TIN2-WT cells, indicating that the lengths of extension by the 47A-hTER telomerase at individual extended telomeres were comparable irrespective of the TIN2 status. These observations argued that the frequency, but not the extension rate, of telomerase extension events was reduced in response to the TIN2-R282H mutation.

### Decreased telomerase recruitment to telomeres in TIN2-R282H cells

**Fig 4:**
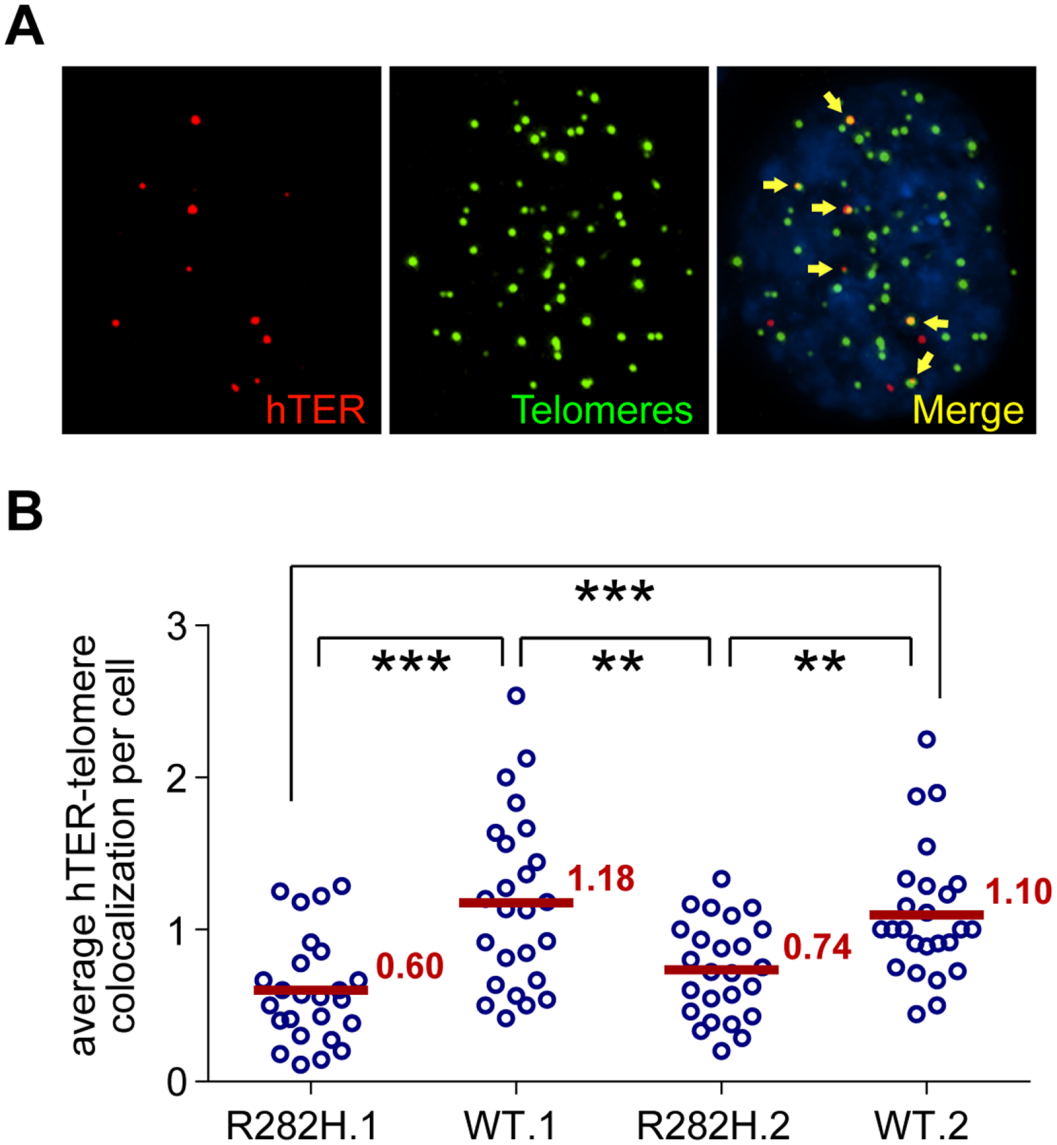
TIN2-R282H mutation reduces colocalization between endogenous telomerase RNA and telomeres. (**A**) Immunostaining-FISH images of WT.1 cells showing co-localization between telomerase RNA and telomeres. Telomeres were identified by immunostaining using a mix of anti-TRF1 and anti-TRF2 antibodies. Telomerase RNA was labeled by RNA FISH. DNA was stained by DAPI. Telomerase RNA spots (red) co-localizing with telomeres (green) are marked by yellow arrows. (**B**) Quantification of telomerase RNA and telomere co-localization in HCT116 knock-in clones. Plot shows the average number of hTER-telomere co-localization per cell. 25 images (~8–15 cells per image) were taken randomly for each cell line at PD8. All quantifications were carried out blindly. Each point on the plot represents value obtained from one image. Mean values are indicated in red. *** (p≤0.001) ** (p≤0.01) calculated by two-tailed Student’s t-tests.

The reduced frequency of telomere extension by the reconstituted 47A-hTER telomerase in TIN2-R282H heterozygotes suggested that telomerase recruitment to telomeres was compromised by the TIN2-R282H mutation. As a final step in our analysis of telomerase function, we directly examined the recruitment of endogenous wild-type telomerase to telomeres by their co-localization in the HCT116 knock-in cells. To do so, cellular localization of telomerase was monitored by RNA-FISH using established oligonucleotide probes complementary to the telomerase RNA component [18,53,54]. A mix of three oligonucleotide probes (~55 nt long) were used, each covalently labeled with five red fluorescence dyes, hence marking one telomerase RNA molecule by as many as fifteen fluorescence dyes, significantly amplifying the signal [55]. We carried out immunofluorescence staining against telomeric shelterin proteins TRF1 and TRF2, followed by FISH for telomerase RNA and analyzed the co-localization between telomerase RNA and telomeres (Fig 4A). Strikingly, we observed that the co-localization between telomerase RNA and telomeres was significantly lower in TIN2-R282H heterozygotes than in TIN2-WT cells (Fig 4B). These observations, combined with those shown in Fig 3, provide direct evidence that the heterozygous TIN2-R282H mutation impairs recruitment of telomerase to telomeres.

## Discussion

In this study, we have identified a novel function for the TIN2 subunit of shelterin, through the analysis of a separation-of-function TIN2 allele recovered from human Dyskeratosis Congenita patients. Using karyotypically stable, telomerase-positive human HCT116 cells, we have generated knock-in clones heterozygous for the DC-associated TIN2-R282H mutation. Notably, the resulting TIN2-R282H heterozygote cells do not display any characteristics of a telomere end protection defect. Instead, two independent systems for interrogating *in vivo* telomerase function at individual telomeres—a mutant repeat incorporation assay and the co-localization of telomerase RNA to telomeres—show that the TIN2-R282H mutation impairs telomerase recruitment, resulting in a reduction of the frequency of telomere extension by telomerase. Our model is consistent with a previous report that ectopically expressed TIN2-R282H pulls down less telomerase activity than wild-type TIN2 [40].

Originally defined of its role in chromosome end protection, the shelterin complex was thought to negatively regulate telomere length by sequestering telomeres away from telomerase. Functional characterization of the shelterin subunit TPP1, however, revealed two surprising roles of TPP1 in promoting telomerase recruitment and telomerase processivity, shifting the view of shelterin as solely an end protection complex which blocks telomerase from acting on telomeres. The work described here further demonstrates that a shelterin-dependent role in promoting telomerase function is not unique to the TPP1 subunit, as revealed by the pronounced telomere shortening in the TIN2-R282H mutant cells. Since TPP1 localization to telomeres is unaffected in TIN2-R282H heterozygotes, this argues that the telomerase recruitment defect of the TIN2-R282H mutant is not conferred through a defect in anchoring TPP1 at telomeres. However, it remains possible that the TIN2-R282H mutation renders TPP1 incompetent for interaction with telomerase. Furthermore, our data show that although telomerase is recruited to telomeres at a reduced frequency in TIN2-R282H heterozygotes, the average length of the extension product at those telomeres that are extended by telomerase is unaffected, suggesting that once telomerase is recruited to telomeres, it is as active as in TIN2-WT cells. Whether telomerase recruitment proceeds through a single coordinated pathway that involves the cooperative behavior of both TIN2 and TPP1, or through independent contributions by these two shelterin subunits, will be a subject for future investigation. For example, whereas TPP1 may have a direct role in recruitment, TIN2 may modify the conformation of telomeres, making them more accessible to telomerase. Interestingly, TIN2 binds to the heterochromatin protein 1γ (HP1γ) through the same region where DC-associated TIN2 mutations cluster. Disrupting TIN2-HP1γ interaction impacts both telomere cohesion and telomere length regulation [22]. HP1γ is required for establishing appropriate sister telomere cohesion and may be involved in shaping the local telomeric chromatin into a more favorable structure for telomerase association.

Of great interest is why the telomere maintenance defects in DC patients carrying heterozygous TIN2 mutations are usually worse than in those carrying heterozygous mutations in the telomerase enzymatic components. Studies of telomerase in induced pluripotent stem cells (iPSCs) have shown that the expression levels of telomerase catalytic subunit and telomerase RNA were both up-regulated significantly during the induction of pluripotency [53,56]. One potential explanation for the more severe form of the disease observed in DC patients carrying TIN2 mutations may be that during early embryonic development, the amount of TIN2, but not the core telomerase components, is the limiting factor for regulating telomerase activity. If so, this also suggests that the recruitment function of TIN2 may be a more tractable target for inhibition of telomerase activity during oncogenesis.

Finally, we point out that our results complement the recent analysis of another TIN2 DC mutation (TIN2-K280E) in knock-in mouse system. This mutation was found to confer both telomerase-dependent and -independent telomere shortening (the exact mechanisms remain to be characterized), as well as cause subtle telomere replication problems [41]. Whether the differences between the two studies are due to a difference in the molecular defect(s) of the two TIN2 DC alleles (TIN2-K280E versus TIN2-R282H) and/or to the differences in telomere maintenance between the two systems (normal mouse cells versus human cancer cells) remains to be determined.

## Materials and Methods

### Construction of donor template

The targeting construct (S1B Fig) was assembled by combining the following segments through overlapping PCR: a 2.7kb genomic fragment containing the human *TINF2* gene, 1.8kb DNA fragment containing the puromycin N-acetyltransferase expression cassette flanked by loxP sites, and 1.8 kb of 3’ flanking DNA of the human *TINF2* gene. MluI sites were engineered at the 5’ and 3’ ends of the construct to clone it into the pBluescript SK vector. Site-directed mutagenesis was used to engineer the silent mutations at the ZFN binding region (5’-CCATGCCAGACCCTGGGGGGAAGGGCTCTGAAG-3’ to 5’-CCTTGCCAGACACTGGGAGGCAGAGCTCTGAAG-3’). For the R282H targeting construct, site-directed mutagenesis was used to introduce the R282H (5’-GAGCGCCCC-3’ to 5’-GAGCACCCC-3’) mutation in exon 6. The ZFN recognition site is ~160bp from the R282H mutation. Full length sequences of the targeting constructs (~6.3kb) were confirmed by DNA sequencing before use.

### ZFN-mediated homologous recombination

TIN2 exon 6-targeting heterodimeric ZFNs (named T2-X6-L5+R4) were expressed in pCMV-FokI(DA+RV) plasmid system. The specificity and efficiency of the ZFNs were described in [43]. 2.5x10^5^ HCT116 cells were plated in 6-well plate 24 hours before transfection. Cells were transfected with 4μg of linearized donor plasmid and 0.5μg of each ZFN encoding plasmid using 10μl of JetPrime transfection reagent (Polyplus). Puromycin selection was applied two days after transfection. Individual colonies were then picked and expanded.

### Screening of knock-in clones

Targeted HCT116 cell clones were screened by Southern blotting of NdeI+KpnI digested genomic DNA. 19 out of 768 clones were identified as correctly targeted clones. PCR analysis was performed on correctly targeted clones using the following primers: F1, 5’-TCTAGCTGGCCGACACTTCAATCT-3’; R1, 5’-CCTGCTAACCCTTTTAGGCACAGC-3’; R2, 5’-CTACCGGTGGATGTGGAATGTGTG-3’. R1 is specific to the unedited allele and R2 is specific to the targeting construct. F1+R1 and F1+R2 PCR products were sequenced to identify clones that contain only the designed change in sequences. PCR was also performed to amplify TIN2 genomic sequences encompassing all coding regions and sequenced to verify no additional mutations were present. To confirm both edited allele and unedited allele of *TIN2* gene were transcribed, RT-PCR analysis was conducted on total RNA using the following primers: RT-F1 (spanning exons 5 and 6), 5’- TGGCTGCTTCCAGAGTGCTCTGTT-3’; RT-R1, 5’- TGGCTTCCTGGCCCTAGGAGGTAA-3’. The target sequence for the TIN2 shRNA is 5’- GAATCCTCCTCAGCAACAA-3’. After the screen procedure, we identified two TIN2-R282H heterozygous clones and four TIN2-WT knock-in clones. All four WT clones maintain stable telomere length over prolonged passaging. Two WT clones that have initial mean telomere lengths comparable to the respective R282H heterozygous clones were then selected for additional telomere maintenance analysis.

### Immunoblotting analysis

Nuclear extracts were made using the NE-PER Nuclear and Cytoplasmic Extraction Reagents (PIERCE). Protein concentrations were determined by performing the Bradford assay (Bio-Rad). Samples were suspended with 2x Laemmli sample buffer, resolved with 10% SDS-PAGE, and detected by Western blotting using the following primary antibodies: mouse anti-TIN2 (Imgenex), mouse anti-TPP1 (Abnova), mouse anti-TRF1 (Genetex), mouse anti-TRF2 (Millipore), rabbit anti-Rap1 (Bethyl), and rabbit anti-POT1 (Abcam ab21382), followed by horseradish peroxidase-conjugated donkey anti-rabbit or anti-mouse IgG (Jackson ImmunoResearch), and visualized by the ECL prime reagent (GE Healthcare). The nuclear protein p84 was detected with a mouse monoclonal anti-p84 antibody (Genetex) as loading controls. Intensities of TIN2 bands were quantified by densitometry using the ImageJ software. Intensities of p84 were used to normalize between different samples.

### Immunoprecipitation analysis

Nuclear extracts were diluted 1:2 in TNE buffer containing 50 mM Tris-HCl (pH 8.0), 150 mM NaCl, 2 mM EDTA, 1% NP-40, and protease inhibitors. The diluted extracts were precleared by incubating with protein G-Sepharose beads (Sigma-Aldrich) at 4°C for 30 min. Immunoprecipitation was carried out by incubating the precleared supernatant with a rabbit polyclonal antibody [8] and protein G-Sepharose beads at 4°C overnight. The beads were washed six times with TNE buffer before fractionation on a 10% SDS-PAGE and Western blotting analysis. 10% of the amount used for the immunoprecipitation was fractionated directly on SDS-PAGE as input.

### Telomere restriction fragment Southern blotting analysis

4–5 μg of genomic DNA was digested with RsaI and HinfI, fractionated by 0.5% agarose gel, then transferred to a Hybond XL membrane and hybridized to an end-labeled telomeric probe (CCCTAA)_4_. Signals were detected by phosphorimaging (Molecular Dynamics). Mean telomere lengths were analyzed by the ImageQuant software. Briefly, the telomere signal intensity over each lane was measured and plotted. The mean telomeric lengths were determined assuming a Gaussian distribution and calculated according to the positions of molecular weight markers run on the same gel.

### Lentiviral plasmids

The pHR’CMV lentiviral expression vector system used in this study was provided by Dr. Didier Trono. Telomerase RNA expressing lentiviral vectors contain the wild-type hTER or 47A-hTER cDNA driven by the IU1 promoter and a GFP gene driven by the CMV promoter [50]. The 47A-hTER template sequence is 3’-CAAACCCAAAC-5’. DN-hTERT- or luciferase- expressing lentiviral vectors contain the DN-hTERT or luciferase cDNA driven by the CMV promoter, followed by an internal ribosome entry site and a hygromycin resistance gene. DN-hTERT contains the D712A and V713I mutations which confer it catalytically inactive [47].

### Lentivirus infection

The day before infection, 2x10^5^ cells were seeded on a 10cm plate and allowed to attach overnight. For viral infection, cells were incubated with virus-containing culture media supplemented with 8μg/ml polybrene. For *in vivo* telomerase function analysis using 47A-hTER, approximately 2 transducing units (TU) of lentivirus per cell were used for each infection. Cells were infected with >90% efficiency as indicated by a GFP expressed from the same lentiviral vector. After 24 hours, the virus-containing media was replaced with fresh media. Infected cells were pooled and passaged for subsequent analysis. For overexpression of DN-hTERT and control luciferase protein, approximately 30 TU of lentivirus per cell were used for each infection.

### Combined immunofluorescence staining and in situ hybridization in interphase cells

For combined immunofluorescence staining-telomere FISH, cells grown on coverslips were fixed with 4% formaldehyde and permeabilized with 0.5% NP40. Immunostaining was carried out by incubating with one of the following primary antibodies: anti-TRF1 (Genetex), anti-TRF2 (Millipore), anti-TPP1 (Abnova), anti-TIN2 (Imgenex), or anti-53BP1 (BD Transduction Laboratories), followed by incubating with secondary antibody conjugated with Alexa Fluor 488 (Molecular Probes). The cells were fixed again with 4% paraformaldehyde and dehydrated by successive incubation in 70%, 95% and 100% ethanol before subjected to telomeric FISH analysis using a TMR-OO-5’-(CCCTAA)_3_−3’ PNA probe as described previously [57]. DNA was stained by 0.1μg /mL DAPI. Coverslips were then mounted onto glass slides in Prolong Gold Antifade Reagent (Invitrogen).

Combined immunofluorescence staining-telomerase RNA FISH was carried out as described [58]. Briefly, cells grown on coverslips were fixed with 4% formaldehyde and permeabilized with 0.1% NP40. Immunostaining was performed by incubating with a mix of anti-TRF1 (Genetex) and anti-TRF2 (Millipore) antibodies to amplify telomere signal, followed by incubating with secondary antibody conjugated with Alexa Fluor 488 (Molecular Probes). The cells were fixed again with 4% paraformaldehyde and dehydrated by successive incubation in 70%, 95% and 100% ethanol. The cells were subsequently rehydrated in 50% formaldehyde in 2XSSC, incubated in prehybridization solution containing 10% dextran sulfate, 50% formamide, 2XSSC, 1mg/ml E. coli tRNA, 1mg/ml RNase-free BSA, 0.5mg/ml salmon sperm DNA, and 2mM vanadyl ribonucleoside complexes. Telomerase RNA FISH was performed by adding a mixture of three Cy3-conjugated telomerase RNA probes (30ng of each per coverslip) [54] to the prehybridization solution and incubating at 37°C in a humidified chamber overnight. The cells were then washed sequentially by 50% formamide in 2XSSC at 37°C, 0.1% NP40 in 2XSSC, 1XSSC and PBS. DNA was stained by 0.2 μg/ml DAPI and the coverslips were mounted onto glass slides in Prolong Gold Antifade Reagent (Invitrogen).

Cell images were acquired with a Nikon Ti-U microscope using a 100x objective and collected as a stack of 0.2 μm increments in the z-axis. After deconvolution using the AutoQuant X3 software, images were viewed with the Maximal Projection option on the z-axis. All image files were randomly assigned coded names to allow blinded scoring for spots co-localization and fluorescence intensity quantification.

### Telomere quantitative FISH on metaphase spreads

Metaphase spreads and telomere fluorescence *in situ* hybridization was performed as described [57], using an Alexa488-OO-5’-(CCCTAA)_3_−3’ and a TMR-OO-5’-(CCCAAA)_3_−3’ PNA probe (Panagene). Images were acquired with a Nikon Ti-U microscope using a 60x objective. All image files were randomly assigned coded names to allow blinded scoring of variant repeats incorporation and fluorescence intensity. Telomere fluorescence intensity was quantified using the ImageJ software. Telomeric variant repeats signals on metaphase chromosomes were segmented manually and the integrated intensity from every segment was quantified. For each metaphase, the average background intensity was determined and subtracted from individual telomere signals.

### Telomerase RNA quantitative real-time PCR

Total RNA was extracted with the TRIzol reagent (Invitrogen). cDNA was prepared using the High Capacity RNA-to-cDNA kit (Invitrogen). Real-time PCR was performed using the Power SYBR green PCR master mix (Invitrogen), with respective set of primers at 50nM concentration, on a StepOnePlus Real-Time PCR machine. Telomerase RNA levels were normalized against GAPDH mRNA levels. Primer sets used: hTER forward 5’- GGTGGTGGCCATTTTTTGTC-3’, hTER reverse 5’-CTAGAATGAACGGTGGAAGGC-3’; GAPDH forward 5’-CATGTTCGTCATGGGTGTGAACCA-3’, GAPDH reverse 5’-ATGGCATGGACTGTGGTCATGAGT-3’.

### TRAP telomerase activity assay

Unless otherwise specified, telomerase activity was analyzed using the TRAPeze kit (Millipore) per manufacturer's directions. The telomeric extension products were separated by 10% TBE-PAGE and visualized by phosphorimaging (Molecular Dynamics).

For 47A-hTER or WT-hTER specific TRAP assay, TRAP reaction was carried out as described in [59] except that the return primer 5’-GCGCGGTACCCATACCCATACCCAAACCCA-3’ was used to detect 47A-hTER activity, and the return primer 5’- GCGCGGTACCCTTACCCT TACCCTAACCCT-3’ was used to detect WT-hTER activity. TRAP products intensity in each lane were quantified by the ImageQuant Software and normalized to the respective internal control intensity.

## Supporting Information

S1 Fig Generation of TIN2-R282H heterozygosity in HCT116 cells. (**A**) Amino acid sequence of the zinc finger modules of the TIN2 exon 6-targeting zinc finger nucleases used in the study. (**B**) Schematic of the gene editing strategy. The two splice variants of TIN2 are shown with the coding regions of exons shaded in gray. 5’ and 3’ arms on the donor template represent regions of homology to the TIN2 genomic sequence. Red asterisk marks the position of the TIN2 mutation. Black asterisk marks the corresponding nucleotide within the TIN2 locus. TIN2 exon 6 ZFN target site is marked by an arrow. Black bar shows the position of Southern blotting probe. Primer mix (F1+R1+R2) were used for genotyping PCR. The bottom panel shows the TIN2 genomic sequence surrounding the ZFN target site. (**C**) Representative image of Southern blotting analysis of HCT116 clones generated using the indicated ZFN pairs together with the donor construct. Genomic DNA was digested with NdeI+KpnI and hybridized with the 3′ probe indicated in (B). Correctly targeted clones are outlined in red. (**D**) PCR genotyping analysis of HCT116 knock-in clones. Clones R282H.1 and R282H.2 carry heterozygous TIN2-R282H mutation. Clones WT.1 and WT.2 carry wild-type TIN2. Genomic DNA was amplified by PCR using primer mix F1+R1+R2. Edited allele gives a band of ~1.4kb by primer pair F1+R2. Unedited allele gives a band of ~1.2kb by primer pair F1+R1. (**E**) Sequence analysis of clone R282H.1 PCR products from (D) showing the G to A mutation on the edited allele (1.4kb product). The unedited allele (1.2kb product) contains the wild-type sequence.
(TIF)

S2 Fig Validating TIN2 expression in HCT116 knock-in clones. (**A**) Schematic of the two isoforms of TIN2 generated by alternative splicing in human cells. Coding regions are shaded in gray. Asterisk marks the position of the R282H mutation. (**B**) RT-PCR analysis on RNA isolated from HCT116 parental cells, TIN2 shRNA-treated HCT116 cells (shRNA targeting sequence marked in (A)), and the HCT116 knock-in clones. Both splice variants of TIN2 were amplified using primer pair RT-F1+RT-R1 (primer positions marked in (A)). Note that the TIN2S splice variant produces longer reverse transcription product. (**C**) Sequence analysis of RT-PCR products from (B) confirms that the TIN2-R282H heterozygotes express both wild-type and mutant TIN2, whereas TIN2-WT clones express wild-type TIN2 only. (**D**) Top panel: immunoblot of nuclear extracts of the HCT116 knock-in clones, HCT116 parental cells, and TIN2 shRNA-treated HCT116 cells. To detect TIN2 protein, we used an antibody (Imgenex) (produced against an N-terminal epitope of TIN2 (a.a. 44–58)) that recognizes both the wild-type and mutant TIN2. Nuclear matrix p84 protein was used as loading control. Bottom panel: levels of TIN2 protein in immuoblots quantified by the ImageJ software, normalized to p84 and relative to that in clone R282H.1 cells. Bars represent mean values of four experiments and SDs. (**E**) Immunoblots of nuclear extracts of the HCT116 knock-in clones for the indicated shelterin proteins. (**F**) The interaction between TIN2 and its shelterin binding partners were examined by immunoprecipitation analysis. Nuclear extracts of the indicated knock-in clones were immnoprecipitated with an anti-TIN2 polyclonal antibody, and probed for TPP1, TRF1 and TRF2 by Western blotting. Asterisk indicates a nonspecific band.
(TIF)

S3 Fig No significant change of telomerase enzymatic activity in TIN2-R282H heterozygotes. (**A**) Levels of telomerase RNA in HCT116 knock-in cells determined by QPCR, normalized to GAPDH and relative to clone R282H.1 cells. Bars represent mean values of three experiments and SDs. (**B**) *in vitro* telomerase activity in HCT116 knock-in clones examined by TRAP assay. Whole cell extracts from 500 and 125 cells were analyzed for each knock-in clone. H: extract treated by heat; IC: internal PCR control.
(TIF)

S4 Fig Low levels of 47A-hTER expression were tolerated well by HCT116 knock-in clones. (**A**) Relative cell number of HCT116 knock-in clones when they were collected for telomere FISH analysis. Same numbers of cells from each knock-in clone were seeded onto plates for lentiviral infection. Cells were infected with an empty lentiviral vector or lentivirus expressing 47A-hTER. Approximately 2 transducing units (TU) of lentivirus per cell were used to infect cells to achieve a 1:1 expression of 47A-hTER: endogenous-hTER. 8 days after infection, parallel cultures of cells were collected for metaphase spreads followed by telomeric FISH, for quantitative PCR, and for counting of cell numbers. Bar graph depicts the cell number of each clone relative to that of R282H.1 cells infected with a vector control. Mean values of three experiments and SDs are shown. 47A/hTER ratio of “0” represents the cell clones infected with an empty lentiviral vector. 47A/hTER ratio of “1” represents the cell clones infected with lentivirus expressing 47A-hTER at a 1:1 ratio relative to endogenous hTER. (**B**) Population doublings that HCT116 knock-in clones had gone through when they were collected for telomere FISH analysis. The knock-in clones were infected and collected as described in (A). Viable cells were counted by hemocytometer and numbers of population doublings were calculated.
(TIF)

S5 Fig Validation of TRAP assay conditions for detecting WT-hTER-containing telomerase or 47A-hTER-containing telomerase. VA13 cells, which are devoid of telomerase catalytic subunit (hTERT) and RNA subunit (hTER), were infected with lentivirus expressing hTERT together with lentivirus expressing WT-hTER or 47-hTER. We used the return primer 5’- GCGCGGTACCCTTACCCT TACCCTAACCCT-3’ to detect WT-hTER-containing telomerase activity, and the return primer 5’-GCGCGGTACCCATACCCATACCCAAACCCA-3’ to detect 47A-hTER-containing telomerase activity. Whole cell extracts from 20 and 5 cells were analyzed for TRAP activity for each sample.
(TIF)
