# Supplementary figures and images for: The Shelterin TIN2 Subunit Mediates Recruitment of Telomerase to Telomeres
Source: PLoS Genet. 2015 Jul 31;11(7):e1005410. doi: 10.1371/journal.pgen.1005410 (PMC4521702; doi:10.1371/journal.pgen.1005410)

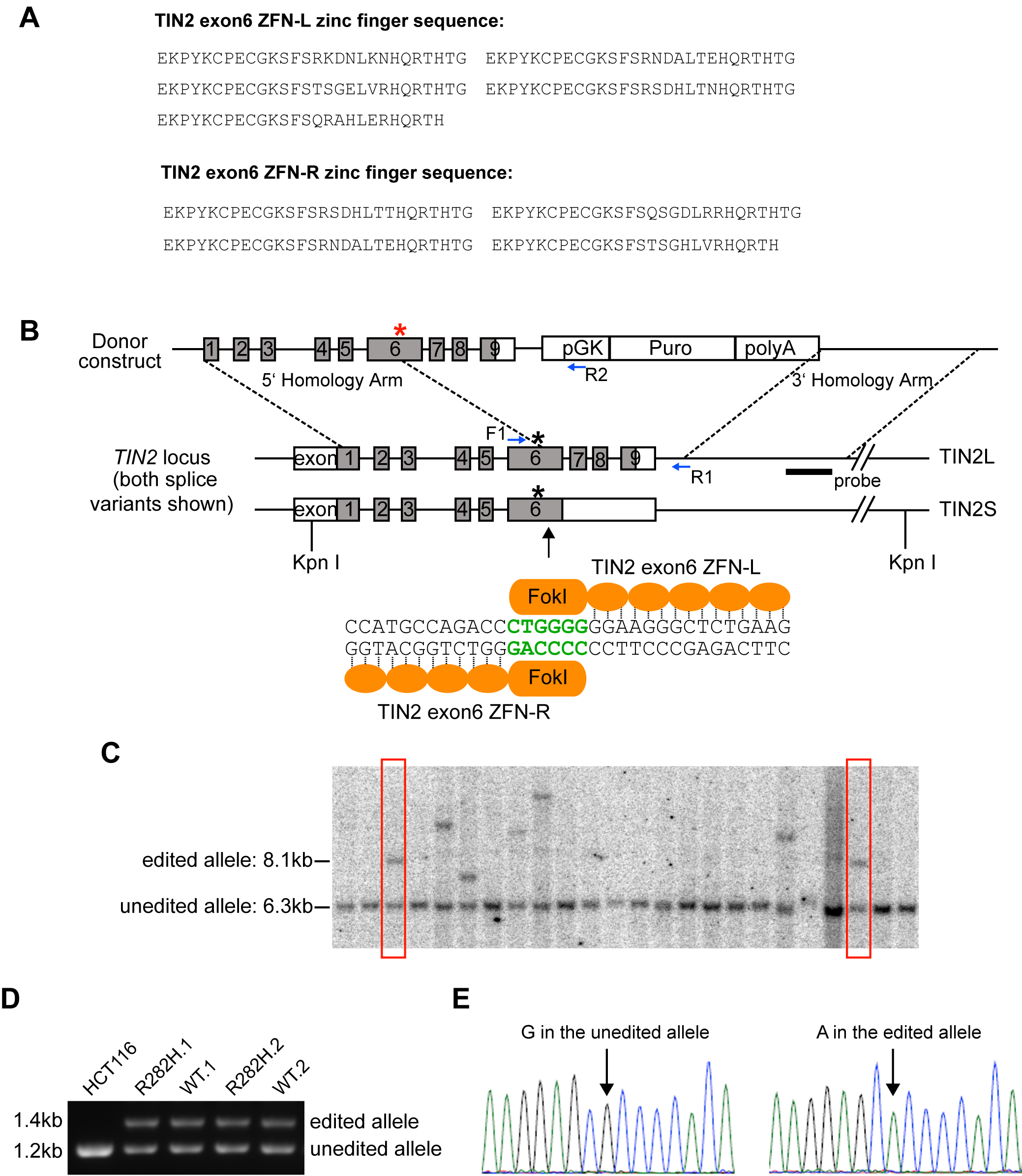

Supplement: S1 Fig — (A) Amino acid sequence of the zinc finger modules of the TIN2 exon 6-targeting zinc finger nucleases used in the study. (B) Schematic of the gene editing strategy. The two splice variants of TIN2 are shown with the coding regions of exons shaded in gray. 5’ and 3’ arms on the donor template represent regions of homology to the TIN2 genomic sequence. Red asterisk marks the position of the TIN2 mutation. Black asterisk marks the corresponding nucleotide within the TIN2 locus. TIN2 exon 6 ZFN target site is marked by an arrow. Black bar shows the position of Southern blotting probe. Primer mix (F1+R1+R2) were used for genotyping PCR. The bottom panel shows the TIN2 genomic sequence surrounding the ZFN target site. (C) Representative image of Southern blotting analysis of HCT116 clones generated using the indicated ZFN pairs together with the donor construct. Genomic DNA was digested with NdeI+KpnI and hybridized with the 3′ probe indicated in (B). Correctly targeted clones are outlined in red. (D) PCR genotyping analysis of HCT116 knock-in clones. Clones R282H.1 and R282H.2 carry heterozygous TIN2-R282H mutation. Clones WT.1 and WT.2 carry wild-type TIN2. Genomic DNA was amplified by PCR using primer mix F1+R1+R2. Edited allele gives a band of ~1.4kb by primer pair F1+R2. Unedited allele gives a band of ~1.2kb by primer pair F1+R1. (E) Sequence analysis of clone R282H.1 PCR products from (D) showing the G to A mutation on the edited allele (1.4kb product). The unedited allele (1.2kb product) contains the wild-type sequence. (TIF) [file pgen.1005410.s001.tif]

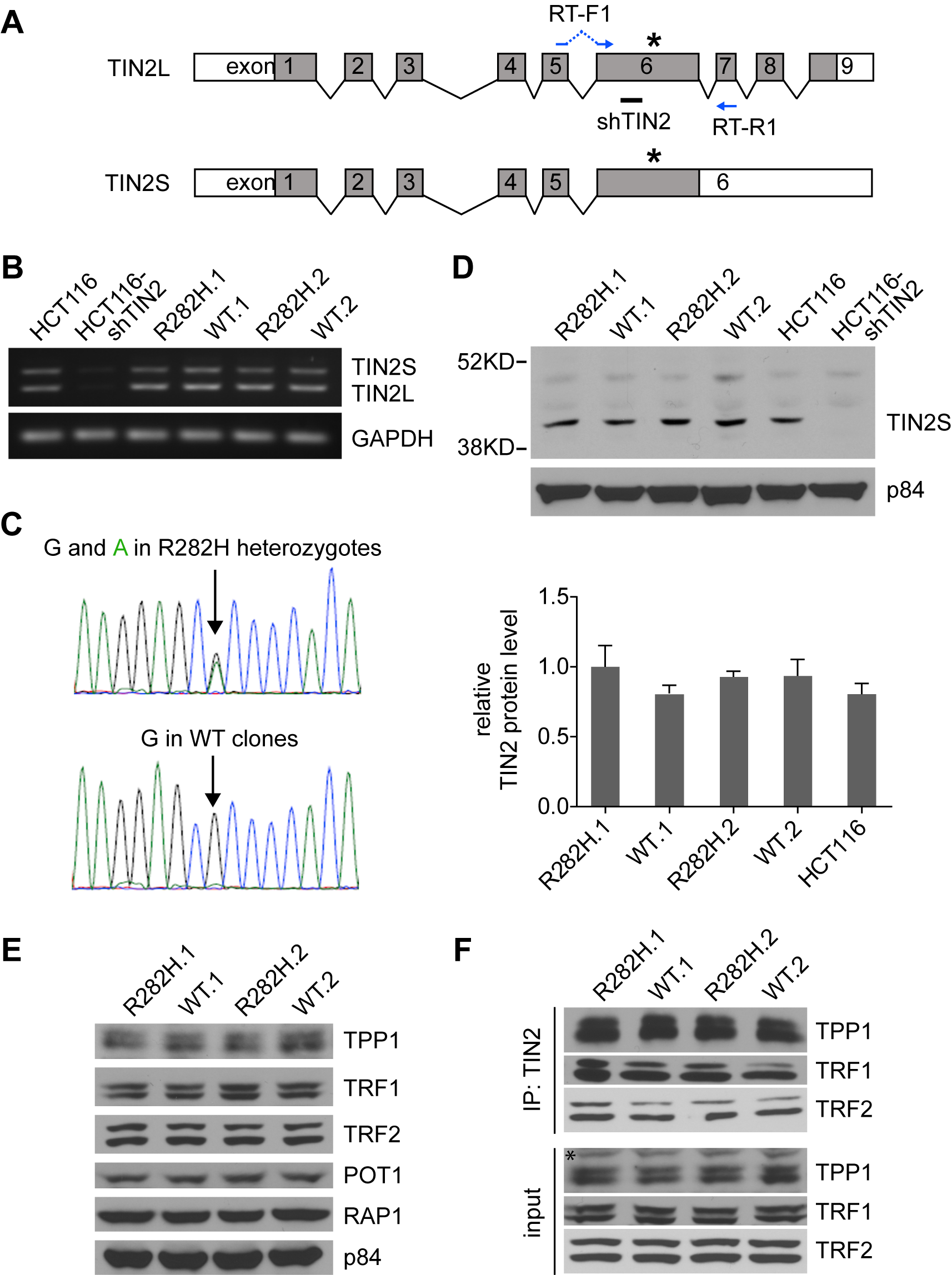

Supplement: S2 Fig — (A) Schematic of the two isoforms of TIN2 generated by alternative splicing in human cells. Coding regions are shaded in gray. Asterisk marks the position of the R282H mutation. (B) RT-PCR analysis on RNA isolated from HCT116 parental cells, TIN2 shRNA-treated HCT116 cells (shRNA targeting sequence marked in (A)), and the HCT116 knock-in clones. Both splice variants of TIN2 were amplified using primer pair RT-F1+RT-R1 (primer positions marked in (A)). Note that the TIN2S splice variant produces longer reverse transcription product. (C) Sequence analysis of RT-PCR products from (B) confirms that the TIN2-R282H heterozygotes express both wild-type and mutant TIN2, whereas TIN2-WT clones express wild-type TIN2 only. (D) Top panel: immunoblot of nuclear extracts of the HCT116 knock-in clones, HCT116 parental cells, and TIN2 shRNA-treated HCT116 cells. To detect TIN2 protein, we used an antibody (Imgenex) (produced against an N-terminal epitope of TIN2 (a.a. 44–58)) that recognizes both the wild-type and mutant TIN2. Nuclear matrix p84 protein was used as loading control. Bottom panel: levels of TIN2 protein in immuoblots quantified by the ImageJ software, normalized to p84 and relative to that in clone R282H.1 cells. Bars represent mean values of four experiments and SDs. (E) Immunoblots of nuclear extracts of the HCT116 knock-in clones for the indicated shelterin proteins. (F) The interaction between TIN2 and its shelterin binding partners were examined by immunoprecipitation analysis. Nuclear extracts of the indicated knock-in clones were immnoprecipitated with an anti-TIN2 polyclonal antibody, and probed for TPP1, TRF1 and TRF2 by Western blotting. Asterisk indicates a nonspecific band. (TIF) [file pgen.1005410.s002.tif]

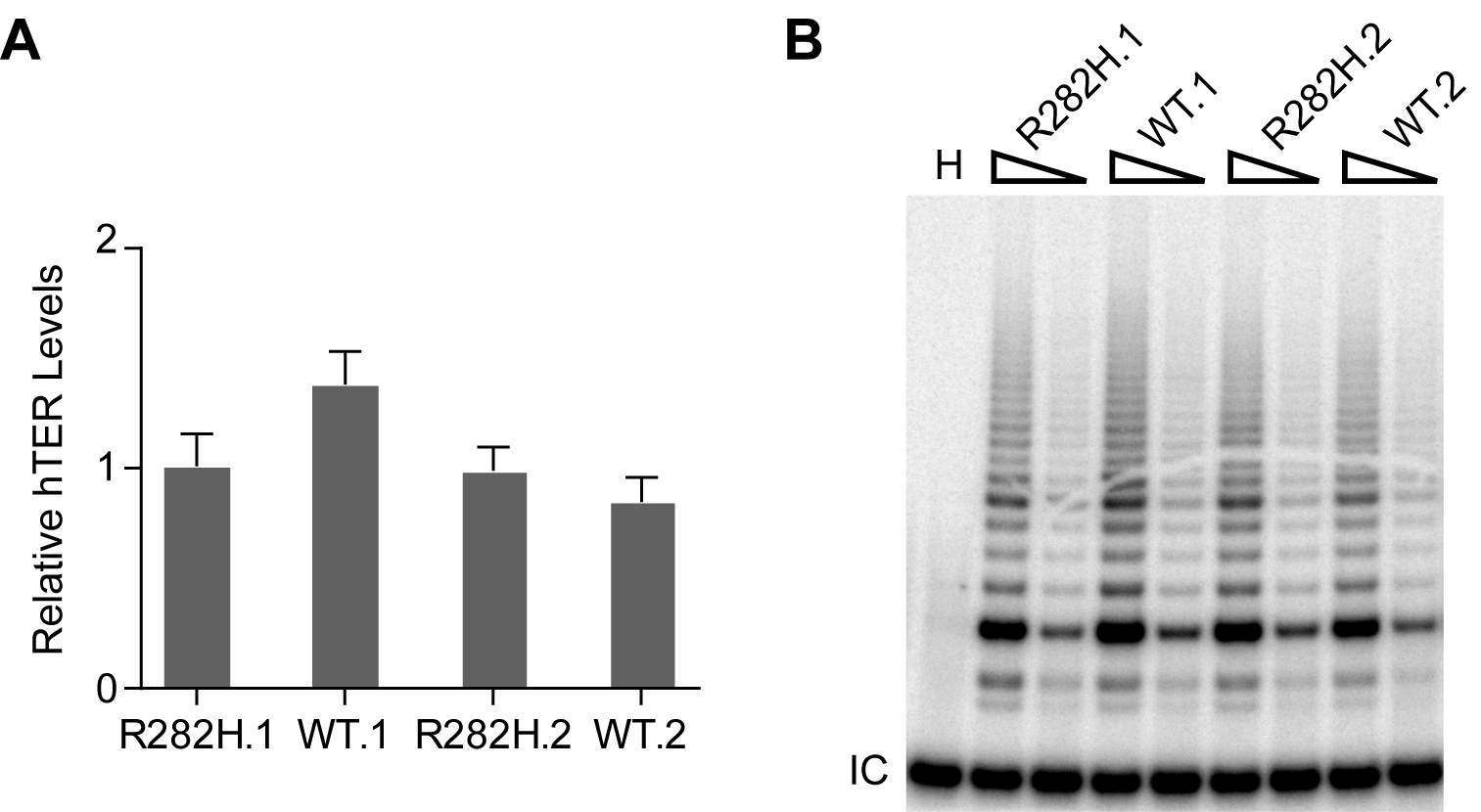

Supplement: S3 Fig — (A) Levels of telomerase RNA in HCT116 knock-in cells determined by QPCR, normalized to GAPDH and relative to clone R282H.1 cells. Bars represent mean values of three experiments and SDs. (B) in vitro telomerase activity in HCT116 knock-in clones examined by TRAP assay. Whole cell extracts from 500 and 125 cells were analyzed for each knock-in clone. H: extract treated by heat; IC: internal PCR control. (TIF) [file pgen.1005410.s003.tif]

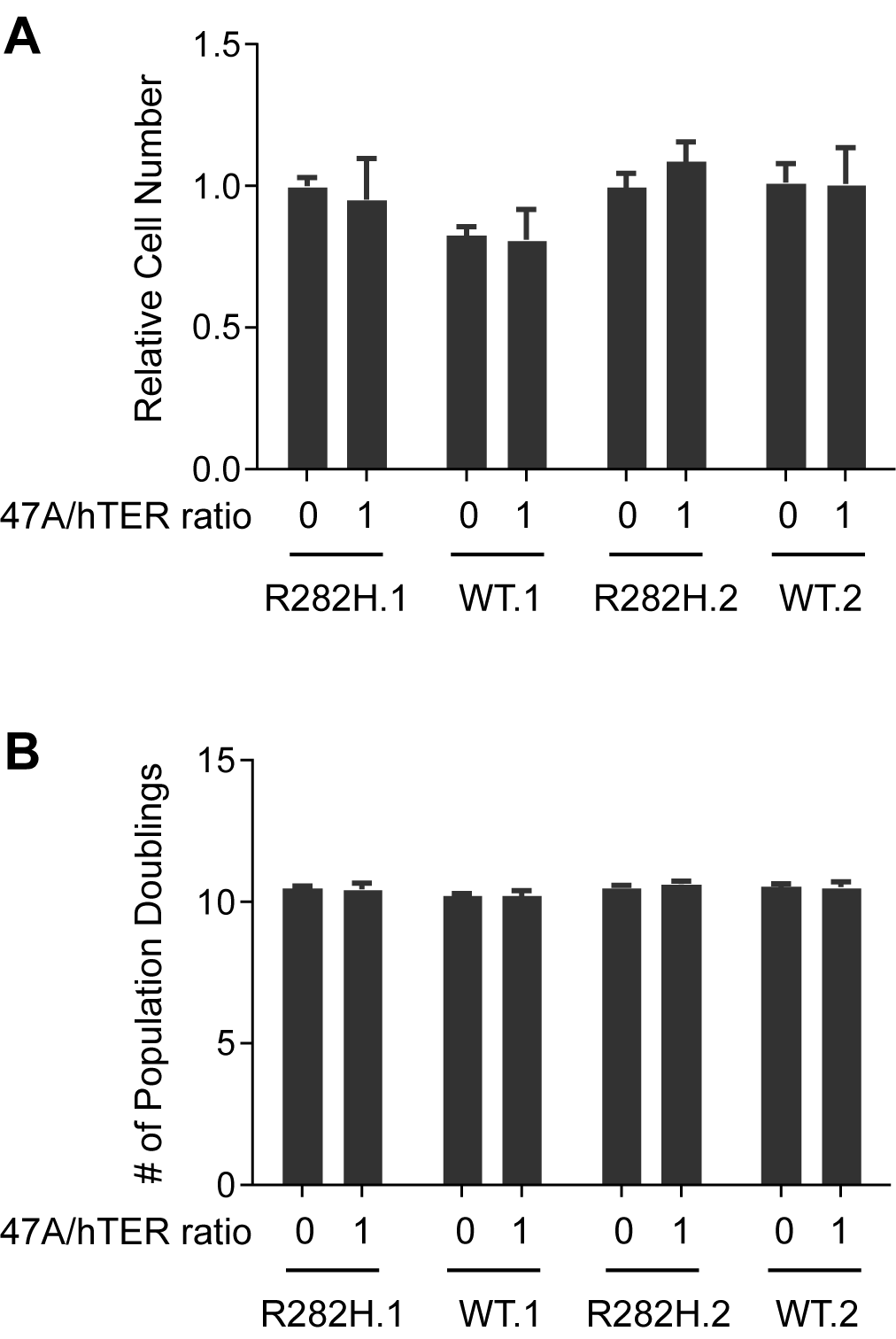

Supplement: S4 Fig — (A) Relative cell number of HCT116 knock-in clones when they were collected for telomere FISH analysis. Same numbers of cells from each knock-in clone were seeded onto plates for lentiviral infection. Cells were infected with an empty lentiviral vector or lentivirus expressing 47A-hTER. Approximately 2 transducing units (TU) of lentivirus per cell were used to infect cells to achieve a 1:1 expression of 47A-hTER: endogenous-hTER. 8 days after infection, parallel cultures of cells were collected for metaphase spreads followed by telomeric FISH, for quantitative PCR, and for counting of cell numbers. Bar graph depicts the cell number of each clone relative to that of R282H.1 cells infected with a vector control. Mean values of three experiments and SDs are shown. 47A/hTER ratio of “0” represents the cell clones infected with an empty lentiviral vector. 47A/hTER ratio of “1” represents the cell clones infected with lentivirus expressing 47A-hTER at a 1:1 ratio relative to endogenous hTER. (B) Population doublings that HCT116 knock-in clones had gone through when they were collected for telomere FISH analysis. The knock-in clones were infected and collected as described in (A). Viable cells were counted by hemocytometer and numbers of population doublings were calculated. (TIF) [file pgen.1005410.s004.tif]

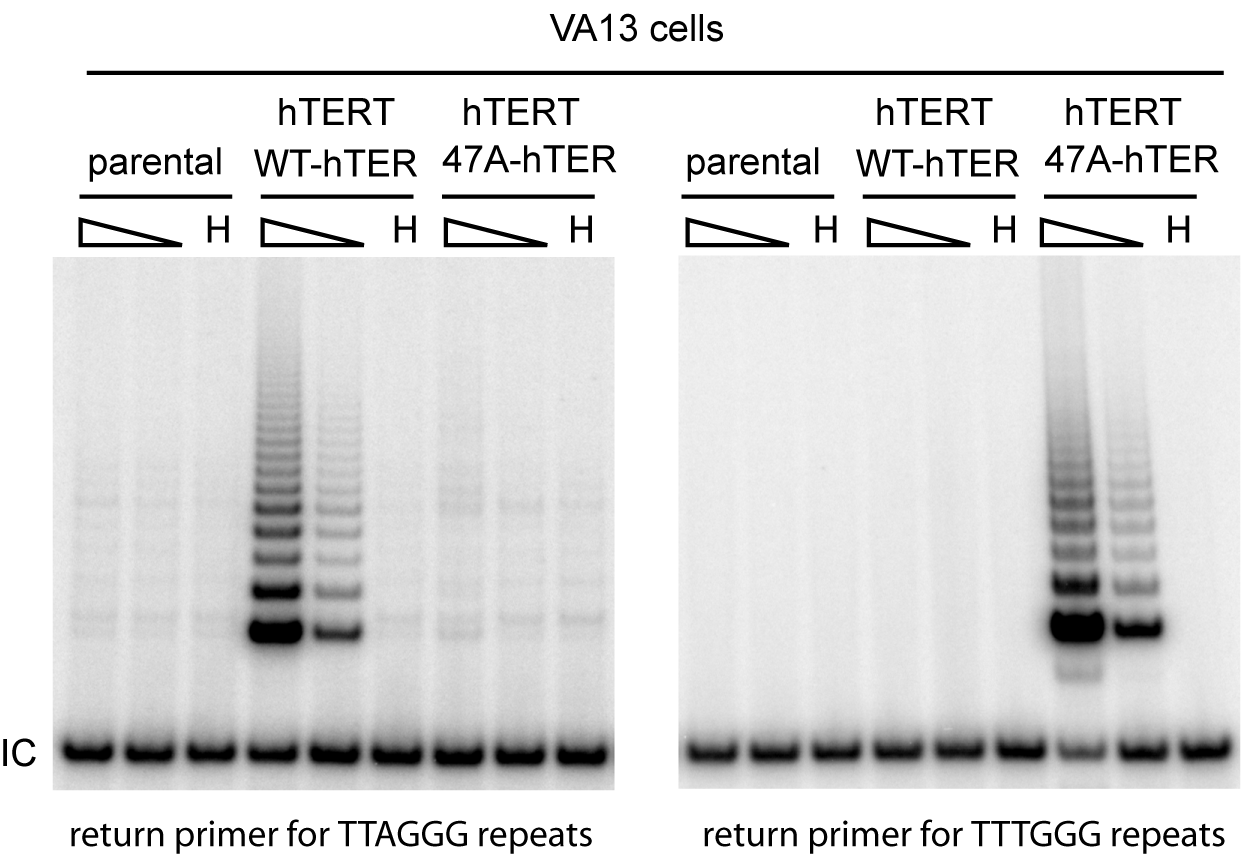

Supplement: S5 Fig — VA13 cells, which are devoid of telomerase catalytic subunit (hTERT) and RNA subunit (hTER), were infected with lentivirus expressing hTERT together with lentivirus expressing WT-hTER or 47-hTER. We used the return primer 5’- GCGCGGTACCCTTACCCT TACCCTAACCCT-3’ to detect WT-hTER-containing telomerase activity, and the return primer 5’-GCGCGGTACCCATACCCATACCCAAACCCA-3’ to detect 47A-hTER-containing telomerase activity. Whole cell extracts from 20 and 5 cells were analyzed for TRAP activity for each sample. (TIF) [file pgen.1005410.s005.tif]
